# Preclinical evaluation of racotumomab, an anti-idiotype monoclonal antibody to N-glycolyl-containing gangliosides, with or without chemotherapy in a mouse model of non-small cell lung cancer

**DOI:** 10.3389/fonc.2012.00160

**Published:** 2012-11-08

**Authors:** Valeria I. Segatori, Ana M. Vazquez, Daniel E. Gomez, Mariano R. Gabri, Daniel F. Alonso

**Affiliations:** ^1^Laboratory of Molecular Oncology, Department of Science and Technology, Quilmes National UniversityBuenos Aires, Argentina; ^2^Center of Molecular Immunology, Innovation Managing DirectionLa Habana, Cuba

**Keywords:** cancer immunotherapy, anti-idiotype antibody, N-glycolylneuraminic acid, NSCLC, mouse models

## Abstract

N-glycolylneuraminic acid (NeuGc) is a sialic acid molecule usually found in mammalian cells as terminal constituents of different membrane glycoconjugates such as gangliosides. The NeuGcGM3 ganglioside has been described as a tumor antigen for non-small cell lung cancer (NSCLC) in humans. Racotumomab is an anti-NeuGc-containing gangliosides anti-idiotype monoclonal antibody (mAb) (formerly known as 1E10) that has received attention as a potential active immunotherapy for advanced lung cancer in clinical trials. In this work, we have examined the antitumor activity of racotumomab in combination or not with chemotherapy, using the 3LL Lewis lung carcinoma as a preclinical model of NSCLC in C57BL/6 mice. Vaccination with biweekly doses of racotumomab at 50–200 μg/dose formulated in aluminum hydroxide (racotumomab-alum vaccine) demonstrated a significant antitumor effect against the progression of lung tumor nodules. Racotumomab-alum vaccination exerted a comparable effect on lung disease to that of pemetrexed-based chemotherapy (100 mg/kg weekly). Interestingly, chemo-immunotherapy was highly effective against lung nodules and well-tolerated, although no significant synergistic effect was observed as compared to each treatment alone in the present model. We also obtained evidence on the role of the exogenous incorporation of NeuGc in the metastatic potential of 3LL cells. Our preclinical data provide support for the combination of chemotherapy with the anti-idiotype mAb racotumomab, and also reinforce the biological significance of NeuGc in lung cancer.

## Introduction

N-glycolylneuraminic acid (NeuGc) is a sialic acid molecule usually found in mammalian cells as terminal constituents of different membrane glycoconjugates such as the GM3 ganglioside (NeuGcGM3). Gangliosides are a broad family of glycosphingolipids found on the outer cell membrane, involved in cell communication, regulation of the immune response, and cancer progression (Patra, 2008; Lopez and Schnaar, 2009). NeuGcGM3 has been described as a tumor antigen for non-small cell lung cancer (NSCLC) in humans (van Cruijsen et al., 2009; Blanco et al., 2012). The significance of NeuGc overexpression in human cancer is still under investigation. Considering that anti-NeuGc antibodies can be detected in several cancer patients, it was hypothesized that antibody-mediated inflammation could facilitate tumor progression (Varki, 2010). However, it is widely accepted that high titers of these antibodies can induce tumor cell death (Roque Navarro et al., 2008; Varki, 2010; Hernandez et al., 2011). In addition, experimental data indicated that growth-stimulating features of NeuGc on tumor cells can be explained by immune system down-modulation (De Leon et al., 2008).

Racotumomab is an anti-NeuGc-containing gangliosides anti-idiotype monoclonal antibody (mAb), formerly known as 1E10, that has received attention as a potential active immunotherapy for advanced lung cancer in clinical trials (Neninger et al., 2007; Alfonso et al., 2008). As an anti-idiotype antibody, racotumomab is the mirror image of the P3 mAb idiotype which specifically reacts against NeuGc antigens on cell surface (Vazquez et al., 1998).

Previously, we evaluated the antitumor activity of racotumomab in syngeneic mouse tumor models. Vaccination with several biweekly intraperitoneal doses of racotumomab coupled to keyhole limpet hemocyanin in Freund's adjuvant, significantly inhibited the formation of spontaneous lung metastases by F3II mammary carcinoma cells (Vazquez et al., 2000). Administration of low-dose cyclophosphamide together with subcutaneous immunization with aluminum hydroxide-precipitated racotumomab (racotumomab-alum vaccine) significantly reduced F3II primary tumor growth. The antitumor response was comparable to that obtained with standard high-dose chemotherapy in such breast cancer model, but without overt signs of toxicity. Interestingly, combinatory chemo-immunotherapy promoted CD8^+^ lymphocyte tumor infiltration and increased tumor apoptosis (Fuentes et al., 2010). In addition, intravenous administration of uncoupled racotumomab, as a biological response modifier, dramatically inhibited metastatic lung colonization by B16 melanoma cells (Vazquez et al., 2000).

In the present work, we have examined the antitumor activity of racotumomab-alum in combination or not with pemetrexed- or taxane-based chemotherapy, using the 3LL Lewis lung carcinoma in C57BL/6 mice as a preclinical model of NSCLC. It is a validated model for the NeuGcGM3 ganglioside, showing an increased expression of such specific antigen in disseminated nodules with respect to the primary tumor or *in vitro* cultured cells (Labrada et al., 2010). In this regard, we also obtained evidence on the role of the exogenous incorporation of NeuGc in the metastatic potential of 3LL cells.

## Materials and methods

### Racotumomab-alum vaccine

Racotumomab was produced by the Center of Molecular Immunology (La Habana, Cuba). The mAb was purified from mouse ascites by good manufacturing practices, as previously described (Alfonso et al., 2002). Briefly, purification was performed by DEAE-exchange chromatography followed by affinity chromatography and size exclusion chromatography using a Sephadex G-25 column. The vaccine preparation was produced by mixing aluminum hydroxide as adjuvant with purified racotumomab at a final concentration of 1 mg/ml. Some experiments were carried out using a bioreactor-obtained mAb, as recently described by Machado et al. (2011).

### Tumor cells and culture conditions

We used the 3LL Lewis lung carcinoma, clone D122, a low immunogenic and high-metastatic cell line in syngeneic C57BL/6 mice (Eisenbach et al., 1984). Additionally, the X63 murine myeloma cell line, expressing high levels of NeuGcGM3 in its membranes, was employed. Tumor cells were maintained in Dulbecco's Modified Eagle Media (DMEM) culture medium (Gibco BRL, Carlsbad, CA, USA) containing 10% heat-inactivated fetal bovine serum. Cells were subcultured twice a week using trypsin-EDTA, and cell viability was assessed using the trypan blue exclusion technique. The concentration of chemotherapy drug causing 50% growth inhibition (IC_50_) was determined by the MTT colorimetric assay.

### Animals

Pathogen-free C57BL/6 mice (approximately 10 weeks-old, with an average weight of 25 g) were obtained from the Animal Care Division of UNLP (La Plata, Argentina). Up to 5–6 mice per cage were kept with water and food *ad libitum* in the animal house facility at Quilmes National University. Pooled sera from experimental or control groups were obtained, and frozen at −20°C in aliquots for further analysis. Experimental protocols were approved by the Animal Review Board and maintenance of animals was conducted under accepted international standards.

### *Ex vivo* NeuGc preincubation

Tumor cells were harvested with trypsin-EDTA solution and resuspended in serum-free DMEM containing NeuGc (Sigma-Aldrich, St. Louis, MO, USA) at a final concentration of 100 μg/ml. After an incubation of 1 h at 37°C, 3LL cells were extensively washed and resuspended in fresh culture medium.

### NeuGcGM3 detection by flow cytometric assay

We used the specific anti-NeuGcGM3 mouse IgG1 mAb 14F7 (Carr et al., 2000), produced by the Center of Molecular Immunology. Tumor cells were harvested with trypsin-EDTA solution, resuspended in serum-free DMEM, and 0.5–1 × 10^6^ cells per sample were incubated with 2 μg of 14F7, isotype control, or mouse sera (dilution 1:50) for 30 min at room temperature. Then, tumor cells were washed with phosphate buffered saline and incubated with R-phycoerythrin-conjugated goat anti-mouse immunoglobulins (DakoCytomation, Carpinteria, CA, USA) for 30 min at 4°C. A total of 5 × 10^4^ events were analyzed per tube with a FACScan flow cytometer (Becton Dickinson, San Jose, CA, USA), using the WinMDI 2.9 software.

### Primary tumor growth and spontaneous metastases

At day 0, groups of at least six mice were inoculated subcutaneously in the right flank with 3LL cells (4–5 × 10^5^ viable cells per mouse in 0.2 ml of DMEM). Primary tumor development was monitored by palpation. The largest perpendicular tumor diameters were measured with a caliper thrice a week, and tumor volumes were calculated using the formula π/6 × length × width^2^. Animals were sacrificed by cervical dislocation at day 50 or when subcutaneous tumor volume exceeded 3,000 mm^3^. Lungs were fixed in Bouin's solution and surface lung nodules were counted under a dissecting microscope, as described elsewhere (Alonso et al., 1996). Four doses of 50 μg of racotumomab-alum vaccine were administered s.c. in the interescapular area at 14-day intervals, beginning the day tumor cell inoculation (days 0, 14, 28, and 42). Control animals received only the saline vehicle. When tumors became palpable at day 10–12, mice received 3 weekly i.p. doses of pemetrexed (100 mg/kg) or docetaxel (20 mg/kg).

### Experimental lung metastases

At day 0, groups of at least eight mice were injected into the lateral tail vein with control or NeuGc-preincubated 3LL cells (7.5 × 10^4^ viable cells per mouse in 0.3 ml of DMEM). At day 21, animals were sacrificed and surface lung nodules were counted, as described above. Mice were vaccinated with racotumomab-alum at 50 or 200 μg/dose, receiving 2 doses before (days −14 and −7) and the third after (day +7) tumor cell inoculation.

### Statistical analysis

Statistical analyses were carried out using GraphPad Prism version 3.0 (GraphPad Software, La Jolla, CA, USA).

## Results

### Protection against spontaneous lung tumor formation by immunization with racotumomab-alum

**Figure 1:**
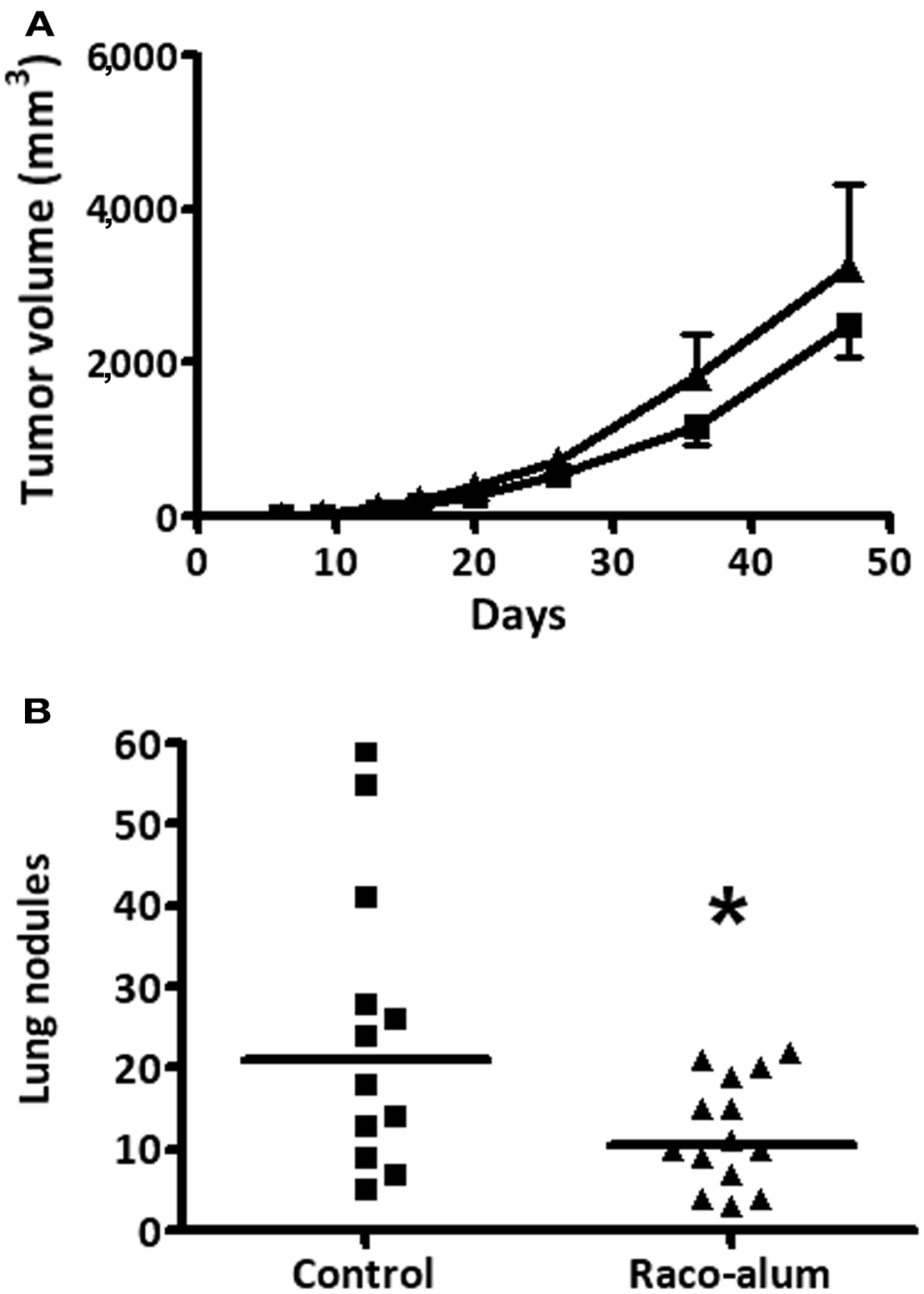
**Antitumor protection against spontaneous lung nodule formation by immunization with racotumomab-alum.** Mice were inoculated subcutaneously with 3LL cells and immunized with racotumomab-alum at a dose of 50 μg, as described in “Materials and Methods.” **(A)** Subcutaneous primary tumor growth. Data represent mean ± SEM. No significant effects were detected between control and treated animals. **(B)** Spontaneous lung tumor nodules. Immunization significantly reduced the number of lung nodules compared to control. Data points represent individual mice and horizontal lines indicate the median values. Data were pooled from two independent experiments with similar results. ^*^p = 0.0377 (unpaired *t*-test with Welch's correction).

We first studied antitumor protection by the anti-idiotype mAb racotumomab against the formation of lung nodules. Tumors were induced in the flank of the mice by subcutaneous injection of 3LL cells and lung lesions were formed by spontaneous metastatic spread. As shown in Figure 1A, growth of subcutaneous primary tumors was not affected by vaccination with biweekly s.c. doses of 50 μg of racotumomab-alum beginning at the day of tumor challenge. On the other hand, immunization significantly reduced the formation of lung tumor nodules (Figure 1B), indicating an increased NeuGcGM3 antigen expression in spontaneous metastatic lesions in comparison to the primary tumor, as previously reported (Labrada et al., 2010). Most experiments were performed using racotumomab produced in mouse ascites fluid. Similar antitumor activity was found using a vaccine formulation containing a bioreactor-obtained mAb within a dose range from 50 to 200 μg per dose, with a reduction of about 40–50% in lung nodule formation (data not shown).

### Combination of anti-idiotype immunization with standard chemotherapy

**Figure 2:**
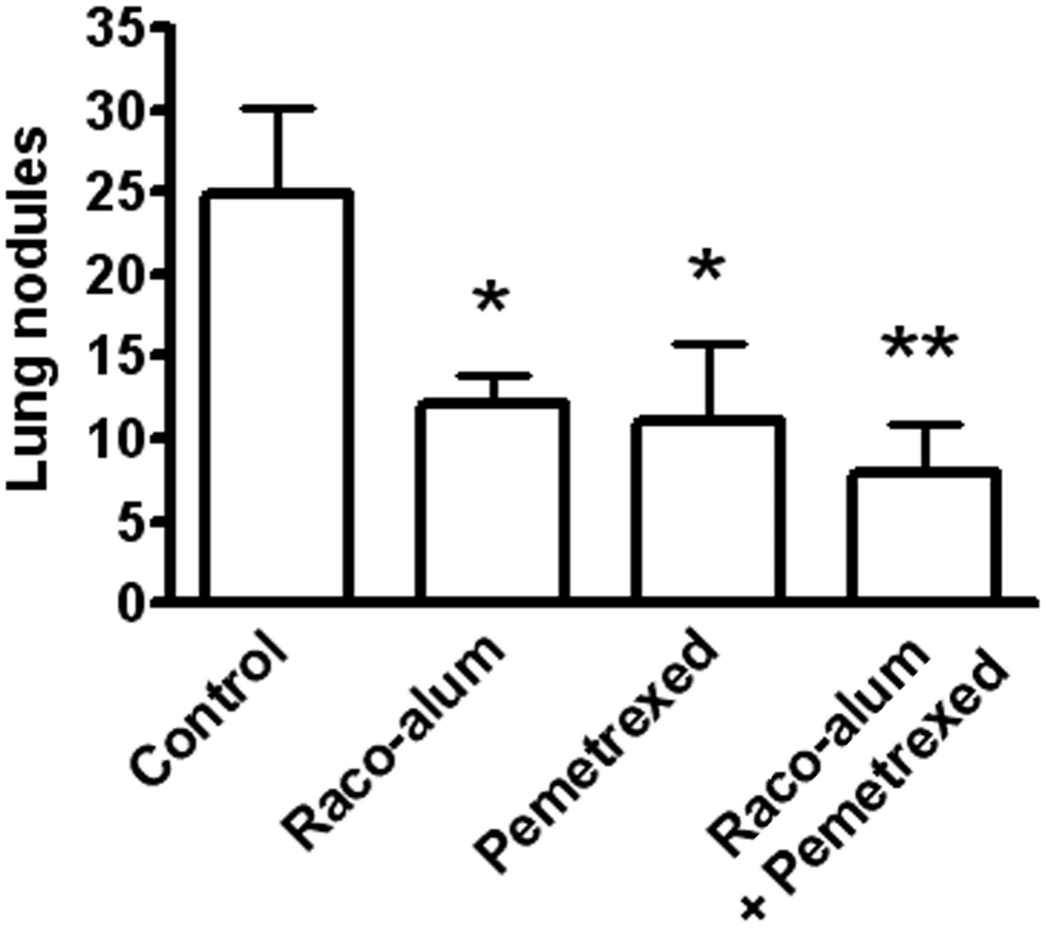
**Effects of racotumomab-alum immunization and chemotherapy on spontaneous lung nodule formation.** Mice were inoculated subcutaneously with 3LL cells, and then immunized with racotumomab-alum (raco-alum) at 50 μg/dose and/or administered with pemetrexed (100 mg/kg), as described in “Materials and Methods.” Data were pooled from two independent experiments with similar results. Results are shown as mean ± SEM. ^*^p < 0.05, control versus raco-alum or versus pemetrexed; ^**^p < 0.01, control versus raco-alum plus pemetrexed (ANOVA followed by Bonferroni's multiple comparison test).

We then explored the antitumor response induced by racotumomab-alum in combination or not with chemotherapy. As racotumomab, pemetrexed had no significant effects on subcutaneous 3LL primary tumors. Immunization with biweekly doses of the vaccine exerted a comparable effect on spontaneous lung tumor formation to that of weekly i.p. cycles of pemetrexed at a dose of 100 mg/kg (Figure 2). Combination of racotumomab-alum with pemetrexed was highly effective against lung nodules (see also Figure 2), although no significant synergistic effect was observed as compared to each treatment alone in the present experimental conditions. No antitumor effects were observed with taxane-based chemotherapy using weekly docetaxel at 20 mg/kg (data not shown). In all cases, chemo-immunotherapy protocols with pemetrexed were well-tolerated, not affecting body weight gain, food and water consumption or inducing other signs of overt toxicity.

### Association of NeuGc expression with highly aggressive experimental lung tumor formation

**Figure 3:**
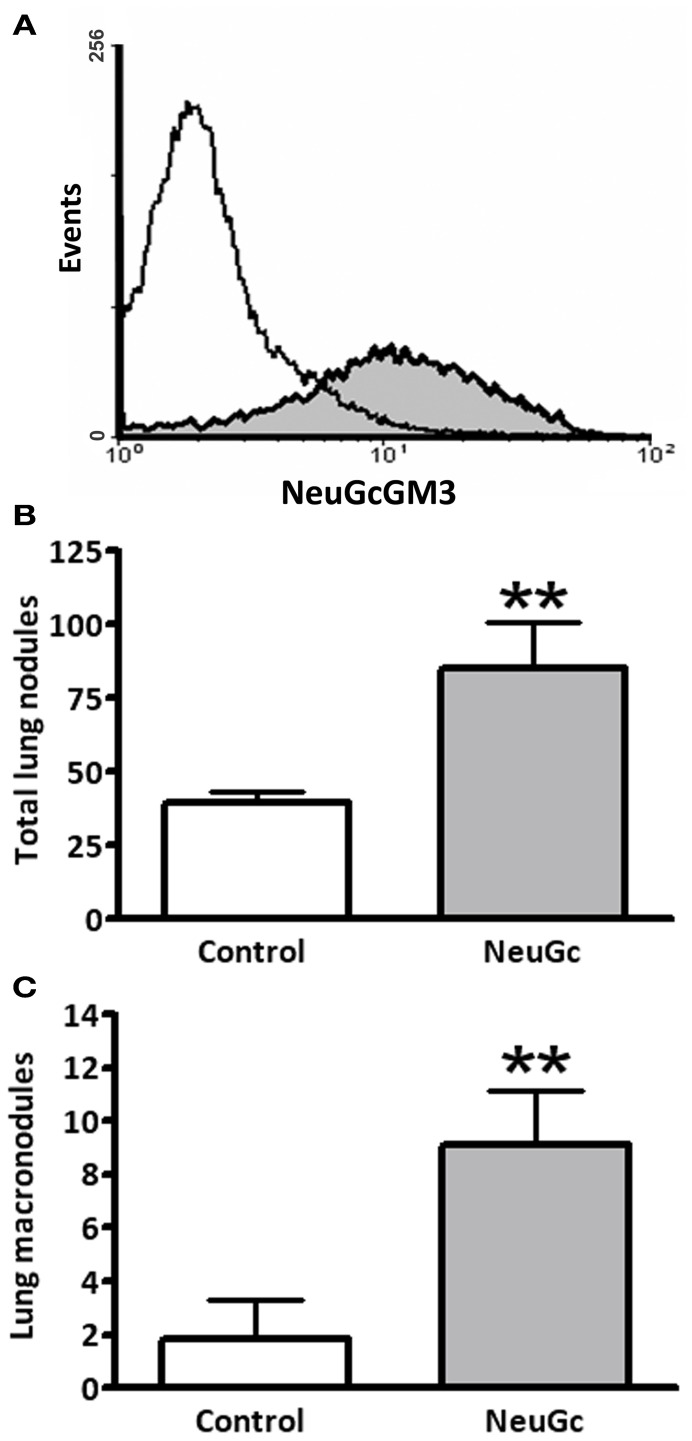
**Effect of exogenous incorporation of NeuGc on experimental lung tumor formation.** 3LL cells were preincubated *in vitro* with purified NeuGc and then injected intravenously in mice, as described in “Materials and Methods.” **(A)** Flow cytometryc analysis of 3LL cells after preincubation with purified NeuGc, resulting in an increase of NeuGcGM3 ganglioside in cell membrane, as detected by the specific 14F7 mAb. White and gray curves present results from control and NeuGc-preincubated cells, respectively. **(B)** Total lung tumor nodules experimentally formed by control or NeuGc-preincubated 3LL cells. **(C)** Experimental formation of lung macronodules (>2 mm in diameter) by control or NeuGc-preincubated 3LL cells. Lung lesions are shown as mean ± SEM. ^**^p < 0.01 (unpaired *t*-test with Welch's correction).

We asked whether antitumor activity of ractumomab was truly associated with expression of NeuGc-containing gangliosides in tumor cells in the present mouse lung-cancer model. We established a highly aggressive experimental disease by intravenous lung colonization by 3LL cells. Lung lesions progressed rapidly and control animals died about 25 days after challenge. In this experimental condition, preliminary experiments demonstrated no antitumor effects of racotumomab-alum, even starting immunization before tumor cell challenge. We hypothesized that during the rapid disease progression, tumor cells are not able to incorporate enough NeuGc from lung tissue, and immunization is not effective. Thus, we decided to perform an *ex vivo* preincubation of 3LL cells with purified NeuGc, following a method known to induce expression of NeuGc gangliosides in the cell membrane (Gabri et al., 2009). To confirm antigen expression, cells were analyzed by flow cytometry with a specific anti-NeuGcGM3 antibody. While a marked staining was detected in NeuGc-preincubated cells, control 3LL cultured cells were almost negative (Figure 3A). We also checked the *in vitro* sensitivity of NeuGc-preincubated tumor cells to chemotherapy. The IC_50_ for pemetrexed after a 3-day exposure of rapidly-growing 3LL cells was about 200 nM (193 ± 6 nM). A similar IC_50_ value was found in 3LL cells preincubated with NeuGc (192 ± 31 nM), suggesting no direct effects of target antigen expression on chemotherapy effectiveness.

**Figure 4:**
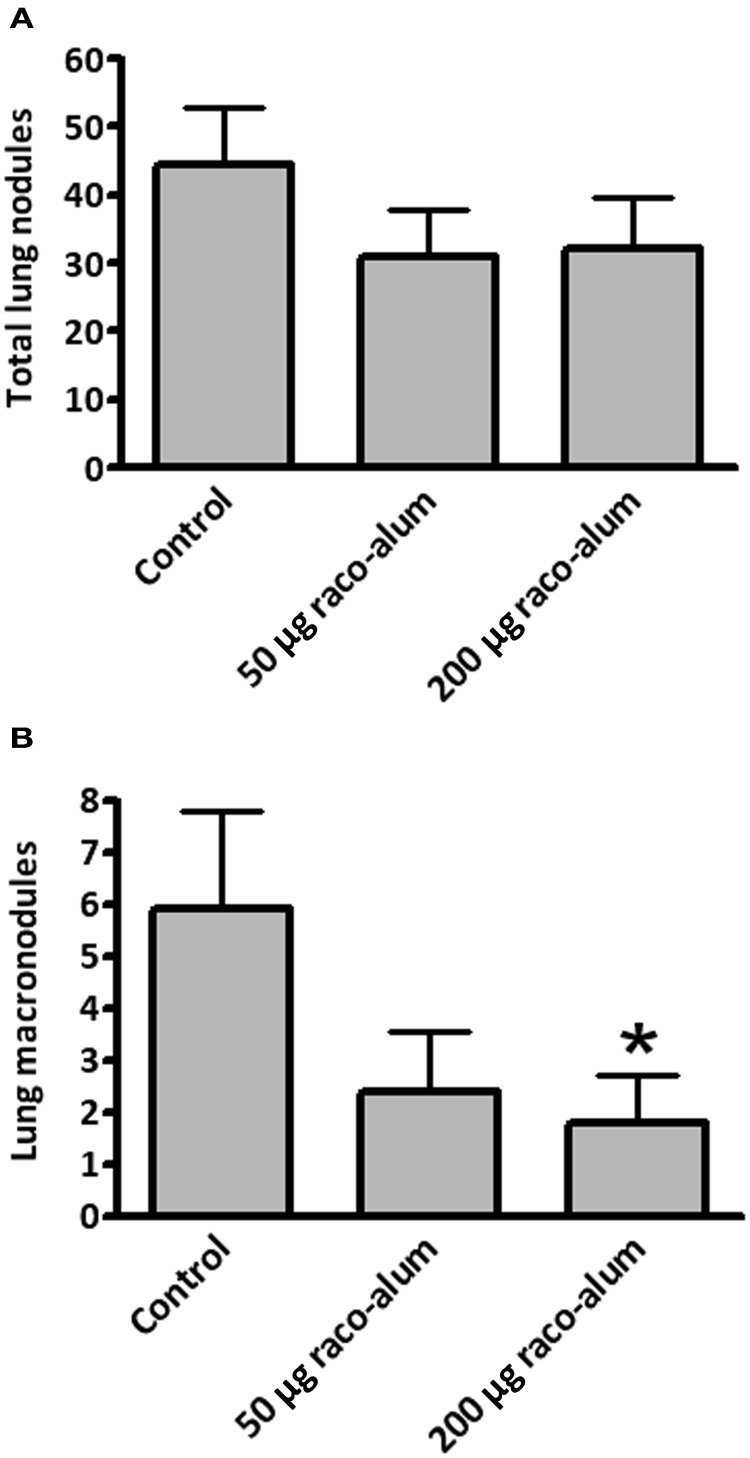
**Antitumor activity of racotumomab-alum vaccination in highly aggressive, NeuGcGM3-positive experimental lung tumors.** Mice were vaccinated with racotumomab-alum (raco-alum) and then injected intravenously with control or NeuGc-preincubated 3LL cells, as described in “Materials and Methods.” **(A)** Total lung tumor nodules. **(B)** Lung macronodules (>2 mm in diameter). Lung lesions are shown as mean ± SEM. ^*^p < 0.05, control versus raco-alum at 200 μg/dose (ANOVA followed by Bonferroni's multiple comparison test).

Preincubation with NeuGc significantly increased the formation of experimental lung tumor nodules, particularly the macronodules of more than 2 mm in diameter (Figures 3B,C). A significant antitumor activity was obtained for macronodules by vaccination with racotumomab-alum at 200 μg per dose in this highly aggressive, NeuGcGM3-positive lung disease (Figure 4).

### Antigen-specific immune response in mice vaccinated with racotumomab-alum

**Figure 5:**
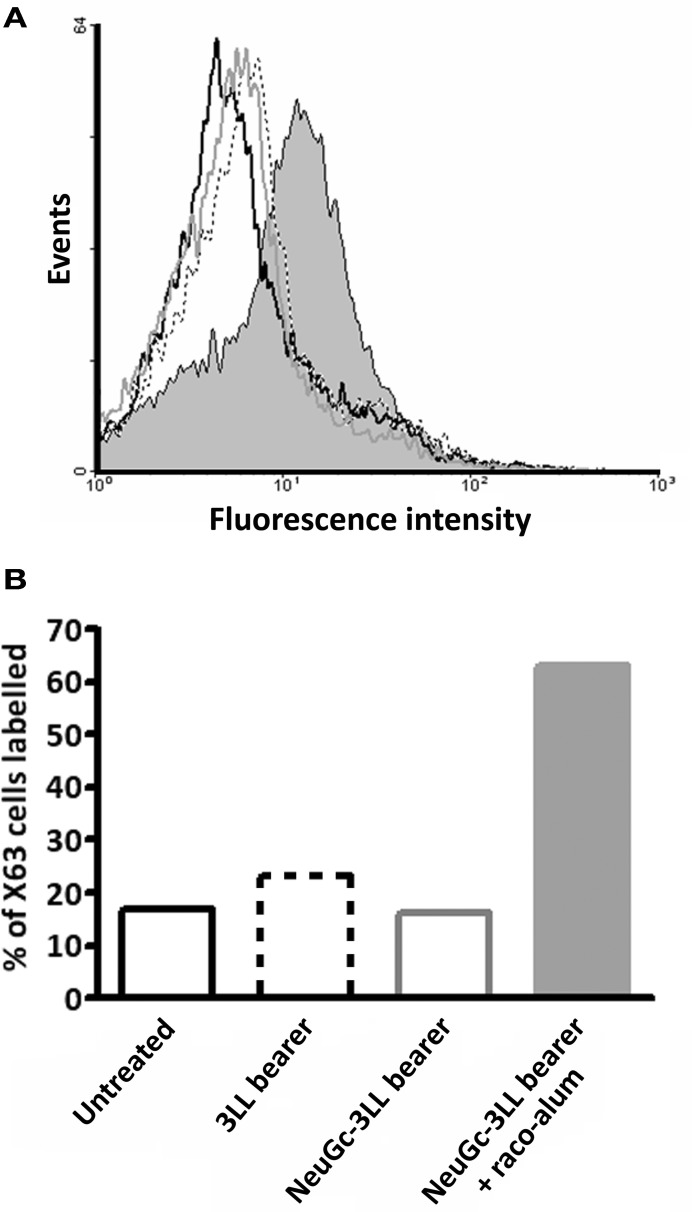
**NeuGc-specific humoral response elicited by racotumomab-alum vaccination.** Mice were challenged subcutaneously with 3LL cells preincubated or not with purified NeuGc, and immunized with racotumomab-alum (raco-alum) at 50 μg/dose, as described in “Materials and Methods.” After tumor growth, pooled sera from the different groups were obtained and analyzed by flow cytometry against X63 cells expressing NeuGcGM3 ganglioside. **(A)** Flow cytometric analysis. **(B)** Percent of recognized X63 cells. Data from healthy untreated mice (black line), mice bearing control 3LL tumors (dotted line), mice bearing NeuGc-enriched 3LL tumors (gray line), and mice bearing NeuGc-enriched 3LL tumors and immunized with raco-alum (filled bar).

Finally, we addressed the NeuGc-specific immune response in mice bearing subcutaneous 3LL tumors. Animals were challenged with 3LL cells preincubated or not with purified NeuGc, and tumors were allowed to progress. Then, the sera were analyzed by flow cytometry using the X63 murine cell line, in which NeuGcGM3 is the major ganglioside expressed on the cell membranes (Hernandez et al., 2008). As shown in Figure 5, no reactivity was observed either in mice bearing NeuGc-enriched or control 3LL tumors. On the contrary, the sera from tumor-bearing animals that were immunized with racotumomab-alum reacted brightly against X63 cells expressing NeuGcGM3 (see also Figure 5).

## Discussion

The development of cancer vaccines, aimed to enhance the immune response against a tumor, is a promising area of research. Preclinical studies would support the development of vaccination strategies with enhanced clinical efficacy (Palena et al., 2006). In this regard, mouse models are excellent tools to test efficacy of novel therapies and to understand the biological mechanisms that govern the generation of an effective antitumor response. One of the main challenges in developing mAb-based cancer therapies directed to glycolipids is to find attractive targets, with specific expression in tumors and a documented role during tumor progression. This is not easy to demonstrate experimentally in murine models, since mouse tumors often do not express similar glycolipids as human tumors do. Besides, human xenograft tumors can grow in immunodeficient athymic mice which are precluded for active immunotherapy studies.

In the present work, we obtained evidence suggesting that exogenous incorporation of NeuGc promotes the metastatic potential of 3LL lung cancer cells and that antitumor activity of the anti-idiotype mAb racotumomab is associated to NeuGcGM3 expression in tumor nodules. Previously, we reported that cultured mouse melanoma and mammary cancer cells are able to process and incorporate NeuGc from different sources such as fetal bovine serum, NeuGc-rich mucins or purified NeuGc, thus promoting the formation of blood-borne metastases (Gabri et al., 2009). NeuGc increased the adhesive properties of tumor cells and seemed to be involved in tumor nesting at distant sites. Similarly, the 3LL Lewis lung carcinoma model was consistent with an increased expression of NeuGcGM3 from subcutaneous primary tumors to spontaneous metastatic lung nodules (Labrada et al., 2010).

In most mammals, the synthesis of NeuGc is catalyzed by the cytidine monophospho-N-acetylneuraminic acid hydroxylase. However, the enzyme is inactivated due to a frameshift mutation in human beings (Irie et al., 1998), and also absent in both human and many mouse cancer cell lines (Segatori et al., 2012). Tumors seem to incorporate NeuGc from dietary sources or the tissue microenvironment. It is known that resistant cancer cells could overexpress NeuGc-containing gangliosides under hypoxic conditions by inducing the sialic acid transporter sialin (Yin et al., 2006).

The ganglioside NeuGcGM3 has been described in several human neoplasms, including NSCLC (van Cruijsen et al., 2009; Blanco et al., 2012), but is usually not detected in healthy human tissues and fluids (Tangvoranuntakul et al., 2003). This fact defines NeuGcGM3 as an interesting neoantigen target for immunotherapy (Fernandez et al., 2010). Assessment of NeuGcGM3 expression in about 200 samples of NSCLC by tissue microarray immunohistochemistry demonstrated a wide expression in more than 90% of cases. Moreover, based on the expression of CD83, which is a marker of mature dendritic cells, NeuGcGM3 appeared to be involved in tumor-induced dendritic cell suppression (van Cruijsen et al., 2009).

A biweekly immunization protocol with racotumomab-alum was highly effective against 3LL lung tumor nodules, either alone or in combination with chemotherapy cycles of pemetrexed (Alimta™). As described previously, the therapeutic effect of racotumomab-alum was associated to an increase of CD4^+^ and CD8^+^ T cell infiltration, a reduced angiogenesis and tumor cell apoptosis in lung nodules, although measurable antibodies were not detected against purified NeuGcGM3 by ELISA assay in C57BL/6 mice (Diaz et al., 2009). Here, we demonstrated that sera from tumor-bearing mice immunized with racotumomab-alum can recognize X63 cells overexpressing NeuGcGM3. It is important to note that sera from non-vaccinated animals bearing 3LL tumors (either with or without NeuGc enrichment) were unable to react with X63 cells indicating that immune response to antigen-positive cells is not elicited by tumor progression itself.

In human NSCLC patients vaccinated with racotumomab, it was suggested a correlation between the induction of IgG or IgM against NeuGcGM3 and longer survival (Hernandez et al., 2008). Anti-NeuGcGM3 specific antibodies were capable of recognizing and killing tumor cells expressing the antigen, by a mechanism resemble the oncotic necrosis (Hernandez et al., 2011).

To our knowledge, this is the first preclinical report demonstrating the feasibility of the combination of the anti-idiotype mAb racotumomab with chemotherapeutic drugs such as pemetrexed, and thus providing a rationale for chemo-immunotherapy combinations in NSCLC. Our experimental data also contribute to reinforce the biological significance of NeuGcGM3 ganglioside as a target for cancer immunotherapy.

### Conflict of interest statement

The authors declare that the research was conducted in the absence of any commercial or financial relationships that could be construed as a potential conflict of interest.
